# Merrimack Valley Dental Association

**Published:** 1864-11

**Authors:** G. A. Gerry


### Proceedings of Societies.

### MERRIMACK VALLEY DENTAL ASSOCIATION.

BY G. A. GERRY.

The annual meeting of the Merrimack Valley Dental Association was held November 3d, at the Council Room, City Hall Building, Manchester, N. H.

After transacting some unimportant business matters, proceeded to the election of officers for the ensuing year, and the following named gentlemen were elected:

A. Lawrence, M. D., of Lowell, *President.*

Dr. D. K. Boutelle, of Manchester, and Dr. E. G. Cummings, of Concord, *Vice Presidents.*

Dr. G. A. Gerry, of Lowell, *Recording Secretary.*

Dr. L. F. Locke, of Nashua, *Corresponding Secretary.*

Dr. S. Lawrence, of Lowell, *Treasurer.*

Dr. G. A. Gerry, of Lowell, *Librarian.*

Drs. C. Heath, of Manchester, D. T. Porter, of Lawrence, A. Lull, of Nashua, S. L. Ward, of Lowell, and E. G. Cummings, of Concord, *Executive Committee.*

Report of Treasurer was read and accepted. Dr. I. J. Wetherbee of Boston, was unanimously elected an honorary member of the Association. Drs. A. Lull of Nashua, and W. P. Kelly of Franklin, were admitted as members. Dr. W. G. Ward, from a committee on Dental Fees, appointed at the last meeting, reported and recommending the adoption by this Association of a uniform tariff of fees, and presented a list for adoption, which for want of time for its proper discussion was laid on the table until the next meeting.

Dr. Locke from a committee appointed to take into consideration the matter of publishing a pamphlet for the education of the people in regard to the teeth reported; which report was accepted, and the committee discharged.

A communication was received from B. S. Cadman, of Boston, stating that he was in no way connected with the American Vulcanite Co., and that his sympathies were entirely with the Dental Profession.

A communication was read from Dr. E. G. Cummings of Concord, and the thanks of the Association were tendered to him for the same.

The following resolution offered by Dr. Gerry was unanimously adopted:

*Resolved*, That it is the duty of every Dental Practitioner to patronize no man or firm, who are connected in any manner with companies, or individuals, who are attempting unjustly, to claim of them remuneration for so called patents.

Dr. Wood introduced the following, which was adopted:

*Resolved*, That the Merrimack Valley Association of Dental Surgeons, heartily approve of the formation of a United States Dental Protective Union, and we pledge it our united support, and recommend it to the favorable consideration of the profession generally.

Dr. I. J. Wetherbee then addressed the Association, explaining the objects of the Dental Protective Union, of which he is the President.

The following essays were then read:

"Professional Egotism," by A. Lawrence, M. D.

Adjourned to meet at Lawrence, Mass., on the first Thursday in May.

